# Mulberry Branch Extracts Enhance the Antioxidant Capacity of Broiler Breast Muscle by Activating the Nrf2 and Cytochrome P450 Signaling Pathway

**DOI:** 10.3390/ani14243702

**Published:** 2024-12-22

**Authors:** Xiang Shi, Wei Qian, Xinlan Wei, Xiaoqing Qin, Jinyan Han, Chao Su, Lijun Bao

**Affiliations:** The Sericultural and Silk Research Institute, College of Animal Science and Technology, Northwest A&F University, Yangling 712100, China; shix0035@163.com (X.S.); qianvii@163.com (W.Q.); wxl18699685076@163.com (X.W.); qinxq3524@163.com (X.Q.); hanjinyan0805@163.com (J.H.)

**Keywords:** broiler breast muscle, mulberry branch extracts, antioxidant capacity, Nrf2 signaling pathway, metabolomics

## Abstract

Mulberry branch extracts (MBEs) have garnered significant attention as natural feed additives and antioxidants; however, their antioxidant properties in meat post-slaughter and their influence on muscle-related metabolic processes remain largely unexplored. Herein, we evaluated the effects of MBEs on the antioxidant capacity and metabolic processes of breast muscle in yellow-feather broilers by adding 0 g/kg, 1.5 g/kg, 3.0 g/kg, and 4.5 g/kg of MBEs to their diets. The results demonstrate that MBEs enhanced the activity of antioxidant enzymes in muscle tissue. Specifically, a real-time quantitative PCR analysis revealed that MBEs increased the expression of antioxidant enzyme genes in a dose-dependent manner, activated the Nrf2 signaling pathway, and upregulated the expression of the Nrf2 gene and its downstream targets at doses of up to 3.0 g/kg. Furthermore, the results of widely targeted metabolomics indicate that the dietary supplementation of MBEs changed the amino acid profile of the muscle, increasing the levels of amino acids and small peptides that contribute to antioxidant properties while reducing the contents of oxidized lipids and carnitine (C5:1) and partially reducing the content of lysophosphatidylcholine (LPC). Notably, at doses of up to 3 g/kg, the levels of five signature bile acids increased in correlation with the added dose. A KEGG analysis indicated that the differential metabolites were predominantly enriched in the metabolism of xenobiotics by cytochrome P450, suggesting that the function of MBEs may be associated with the expression of P450 enzymes. In summary, this study demonstrates that MBEs are effective, safe, and natural antioxidants, offering a viable solution to mitigating oxidative stress in the yellow-feather broiler farming industry and even in livestock farming.

## Simple Summary

This study aimed to conduct a comprehensive analysis of the effects of mulberry branch extracts on antioxidant-related genes, as well as their biological functions and pathways in the breast muscle of yellow-feather broilers. The research was conducted through the integration of antioxidant enzymes, lipid peroxidation products, antioxidant genes, and widely targeted metabolomics. The results demonstrate that the expression levels of both antioxidant enzymes and antioxidant genes increased to varying extents, while the levels of lipid peroxidation products decreased. This suggests the activation of antioxidant-related pathways. Furthermore, a metabolomic analysis identified several pathways associated with differential metabolites, indicating that mulberry branch extracts can influence these pathways to exert antioxidant effects.

## 1. Introduction

Meat plays a crucial role in a healthy and balanced diet, supplying essential nutrients such as protein, fat, vitamins, iron, zinc, and phosphorus [1]. In contemporary society, which increasingly values a “LOHAS” lifestyle, chicken meat has gained widespread popularity among consumers worldwide due to its high-quality protein, its low cholesterol and fat contents, and the lack of religious dietary restrictions surrounding this meat [2]. The consumption of chicken meat has been steadily increasing each year, driven by its affordability and higher content of unsaturated fatty acids and imidazole dipeptides [3]. Furthermore, as economic standards improve, consumers’ demand for food products, including meat, has progressively shifted toward safer, more nutritious, and higher-quality options [4]. In response, the meat industry has been committed to delivering higher-quality meat products to meet these evolving demands [5]. For commercial broilers, which are typically raised in intensive farming systems, the oxidative stress induced by high-density feeding practices and long-distance transportation poses significant challenges to both chicken health and the quality of post-slaughter meat [6,7].

Research has demonstrated that both pre-slaughter stress and post-slaughter storage increase the reactive oxygen species (ROS) content in muscle tissue, negatively affecting the organoleptic quality of the meat. Additionally, both factors also contribute to a partial loss of protein functionality and accelerate amino acid depletion, ultimately diminishing the nutritional value of meat products [8,9]. Therefore, poultry producers must implement novel dietary or technological strategies to mitigate or eliminate oxidative stress, thereby preserving muscle nutrition and enhancing the quality of chicken meat [10,11]. The flavonoids and polyphenols found in mulberry branches possess exceptional antioxidant capacity, with both aqueous and alcoholic extracts demonstrating significant free radical scavenging abilities [12,13]. However, previous studies and applications of mulberry branches have primarily focused on their use as fuel, substrates for mushroom cultivation, or as roughage for animal feed, resulting in a relatively low overall utilization rate of their active ingredients. By comparison, there is a paucity of studies investigating the effects of MBEs on animals, particularly regarding the impact of MBEs on muscle antioxidant defense in yellow-feather broilers. Furthermore, the mechanisms underlying these effects have not been comprehensively explored in the existing literature. Consequently, we evaluated the enhancement of antioxidant capacity in broiler breast muscle through dietary supplementation with MBEs by conducting an integrated analysis of muscle metabolic profiles, antioxidant gene expression, and enzyme activities. This approach clarifies the metabolic levels and molecular foundations that underpin the improvement in meat quality with the dietary addition of MBEs.

## 2. Materials and Methods

### 2.1. Mulberry Branch Extraction Procedure

The spring-felled mulberry branches were sourced from the mulberry field at the Zhouzhi Erqu Experimental Station of Northwest A&F University. The extraction of mulberry branches was carried out by Xi’an Bo’ao Xintian Botanical Development Co., Ltd., (Xi’an, China) following the optimized protocol developed in our laboratory. The specific extraction procedure is as follows: solid–liquid ratio of 1:20, extraction time of 2 h, methanol concentration of 60%, and extraction temperature of 80 °C. The MBEs provided by the company were tested for methanol residues according to the “Determination of Residual Solvents” (0861) method in the 2020 edition of the Chinese Pharmacopoeia, and no methanol was found. The MBEs contain 8.11% total flavonoids, 6.8% crude protein, 2.9% moisture, 2.8% ash, 2.45% total phenols, and 1.10% crude fat.

### 2.2. Animals, Diets, and Sample Collection

A total of 280 male medium-rate yellow-feather broilers, aged 42 days, were randomly divided into four groups: one control group with a basal diet (CK) and three treatment groups receiving the basal diet supplemented with 1.5 g/kg (Treat-1500), 3.0 g/kg (Treat-3000), and 4.5 g/kg (Treat-4500) of the MBEs, respectively. Each group consisted of five replicates, with 14 broilers per replicate. The selected 42-day-old yellow-feather broilers were fed medium-size broiler feed from 42 to 65 days of age, followed by large-size broiler feed from 66 to 90 days of age. The experiment lasted for 49 days, with the nutrient levels of the basal diets (Table 1) based on the National Agricultural Standard for Nutritional Requirements for Yellow-feather Broiler Chickens (NY/T 3645-2020) [14]. At the conclusion of the experiment, after a 12 h fasting period, five broilers from each group were randomly selected. Following this, the broilers were slaughtered. Subsequently, the right pectoral muscles were isolated and stored at −80 °C for further analysis.

### 2.3. Muscle Antioxidative Status Measurements

To evaluate muscle antioxidant indices, we measured the total antioxidant capacity (T-AOC), total superoxide dismutase (T-SOD), catalase (CAT), and glutathione peroxidase (GSH-Px) activities, as well as the malondialdehyde (MDA) levels. These measurements were performed using commercial ELISA kits obtained from the Manheng Biotechnology Development Center (Yangling, China) in accordance with the manufacturer’s instructions. The testing procedures were conducted using an enzyme labeling analyzer (Infinite F50, Tecan Trading AG, Zurich, Switzerland).

### 2.4. Real-Time Quantitative PCR of Key Genes’ mRNA Expression

RT-PCR was employed to assess the expression of antioxidant-related genes. For this, total RNA was extracted from three samples of each group using the Animal Tissue/Cell Total RNA Rapid Extraction Kit and subsequently reverse-transcribed into cDNA using the First Strand Reverse Transcription Kit (gDNA remover). The resulting cDNAs were then analyzed using a BIOER FQD-96A (BORI TECHNOLOGY, Hangzhou, China) for RT-PCR. Specific primers for *β-actin* and antioxidant-related genes (*Nrf2*, *HO-1*, *NQO1*, *GCLC*, *GCLM*, *SOD1*, *CAT*, and *GSH-Px*) were designed based on sequences available in GenBank and synthesized by Sangon Biotech (Shanghai, China). Gene expression was normalized to *β-actin* and calculated using the 2^−ΔΔCt^ method. The primer sequences are provided in Table 2.

### 2.5. Widely Targeted Metabolomics Analysis

Muscle tissue samples stored at −80 °C were sent to Metware for extraction based on standard procedures. The data acquisition system primarily consisted of Ultra Performance Liquid Chromatography (UPLC) (ExionLC AD, https://sciex.com.cn/, accessed on 15 January 2024) and tandem mass spectrometry (MS/MS) (QTRAP, https://sciex.com.cn/, accessed on 15 January 2024). Mobile phase A comprised ultra-pure water with 0.1% formic acid, while mobile phase B consisted of acetonitrile with 0.1% formic acid. Metabolites were eluted stepwise at a flow rate of 0.4 mL/min as follows: 95:5 V/V at 0 min, 80:20 V/V at 2.0 min, 40:60 V/V at 5.0 min, and 1:99 V/V at 6.0 min and at 7.6 min back to 95% A. The column temperature was maintained at 40 °C with an injection volume of 2 μL. Mass spectrometry acquisition conditions included an electrospray ionization (ESI) temperature of 500 °C, mass spectrometry voltages of 5500 V (positive) and −4500 V (negative), ionization gas I (GS I) set at 55 psi, gas II (GS II) at 60 psi, curtain gas (CUR) at 25 psi, and the collision-activated dissociation (CAD) parameter set to high. In a triple quadrupole (Qtrap), each ion pair was scanned for detection using optimized declustering potential (DP) and collision energy (CE). The processed data underwent log transformation and identification based on the Metware database. Bioinformatics analyses were conducted using the ropls R package (4.2.0) on the Metware Cloud Platform (https://cloud.metware.cn/, accessed on 16 June 2024).

### 2.6. Statistical Analysis

Data used in this study were obtained from at least 3 replicates and expressed as means ± standard deviation (SD). All analyses were performed using SPSS 13.0. Differences between groups were conducted using one-way analysis of variance (ANOVA) followed by a Tukey’s post hoc HSD test. Statistical significance was established at *p* < 0.05.

## 3. Results

### 3.1. Effects of MBEs on MDA Content and Antioxidant Enzyme Activities of Broiler Breast Muscle

**Figure 1:**
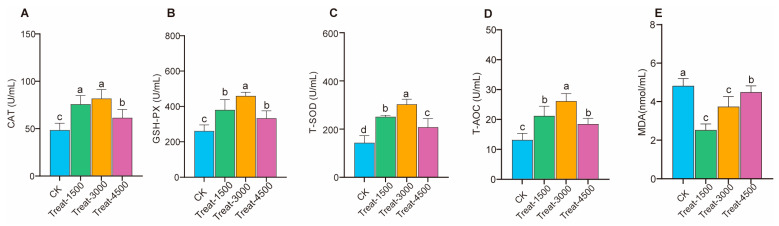
Effect of MBEs on antioxidant capacity in breast muscle of broilers. (**A**) Catalase, CAT. (**B**) Glutathione peroxidase, GSH-Px. (**C**) Total superoxide dismutase, T-SOD. (**D**) Total antioxidant capacity, T-AOC. (**E**) MDA. CK: control group; Treat-1500: basal diet containing 1.5 g/kg; Treat-3000: basal diet containing 3.0 g/kg; Treat-4500: basal diet containing 4.5 g/kg. ^a,b,c,d^ Different letters indicate significant differences for interaction effect (*p* < 0.05).

As shown in Figure 1 and Table S1, the incorporation of MBEs into the diet significantly enhanced T-AOC and the activities of antioxidant enzymes, namely CAT, T-SOD, and GSH-Px, at supplementation levels of 1.5–4.5 g/kg (*p* < 0.05). Additionally, MBE supplementation significantly reduced the content of MDA in broiler breast muscle at addition levels of 1.5–4.5 g/kg (*p* < 0.05). Notably, while T-AOC and the three antioxidant enzymes decreased at the 4.5 g/kg level compared to that at the 3.0 g/kg level, the increases remained significant when compared to the control group.

### 3.2. Antioxidant-Related Gene Expression

**Figure 2:**
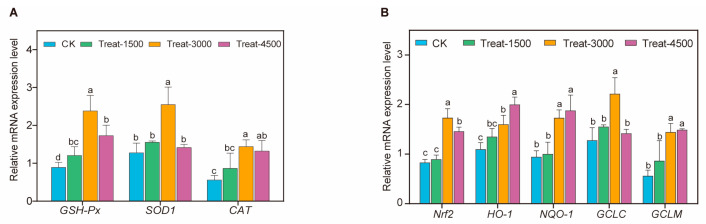
Effects of MBEs on relative mRNA expression levels of antioxidant-related genes. (**A**) Effects of MBEs on relative expression of antioxidant enzyme genes. *GSH-Px*: glutathione peroxidase; *SOD1*: superoxide dismutase 1. *CAT*: catalase. (**B**) Effects of MBEs on relative mRNA expression levels of Nrf2 signaling pathway. *Nrf2*: nuclear factor erythroid 2-related factor 2; *HO-1*: heme oxygenase-1; *NQO-1*: NAD(P)H quinone oxidoreductase-1; *GCLC*: glutamyl–cysteine ligase; *GCLM*: glutamate–cysteine ligase modifier subunit. ^a,b,c,d^ Different letters indicate significant differences for interaction effect (*p* < 0.05).

The effects of dietary supplementation with MBEs on the expression of major antioxidant enzyme genes in broiler breast muscle tissue are presented in Figure 2A (Table S1). At the addition level of 1.5 g/kg, there was no significant effect on the expression of *GSH-Px*, *SOD1*, and *CAT* genes in broiler breast muscle (*p* > 0.05). However, when 3.0 g/kg of MBEs were incorporated into the diet, the expression of *GSH-Px* and *SOD1* genes was highly significantly upregulated (*p* < 0.01), while *CAT* gene upregulation expression was statistically significant (*p* < 0.05). At a supplementation level of 4.5 g/kg, a significant effect was observed on the expression of *GSH-Px* and *SOD1* genes in broiler breast muscle (*p* < 0.05). Moreover, the addition of MBEs to the diet significantly increased the expression of *Nrf2* and its downstream genes (*HO-1*, *NQO-1*, *GCLC*, and *GCLM*) (Figure 2B). Dietary supplementation with 1.5 g/kg of MBEs did not result in significant changes in the expression levels of *Nrf2*, *HO-1*, *GCLC*, and *GCLM* genes in broiler breast muscle (*p* > 0.05). However, when the supplementation level was increased to 3.0 g/kg, the expression of *Nrf2*, *GCLC*, and *GCLM* genes was highly significantly upregulated (*p* < 0.01), while the expression of *HO-1* and *NQO-1* was significantly increased (*p* < 0.05). At the higher supplementation level of 4.5 g/kg MBEs, the expression of *Nrf2*, *HO-1*, *NQO-1*, and *GCLM* was extremely significantly increased (*p* < 0.01), whereas the expression of *GCLC* did not show a significant difference (*p* > 0.05).

### 3.3. The Effects of the Addition of MBEs on the Metabolism of Broiler Breast Muscle

#### 3.3.1. Full Mass Spectrometric and Dynamic Changes in Metabolites in Broiler Breast Muscle

**Figure 3:**
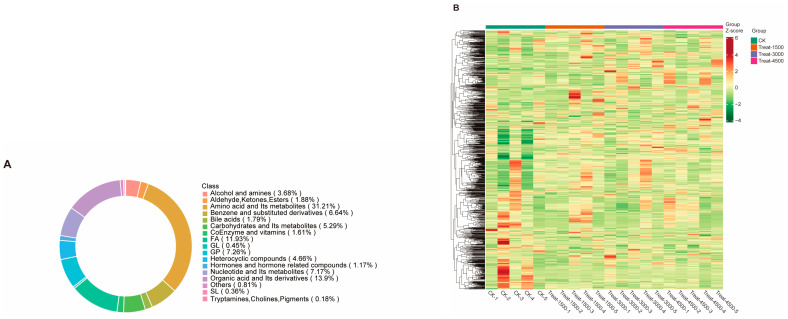
Effect of MBEs on overall metabolites in broiler breast muscle. (**A**) Ring chart of proportion of all metabolite classes. (**B**) Heat map analysis of all metabolites.

In this study, a metabolomics analysis was conducted on muscle samples collected from various groups supplemented with varying amounts of MBEs (0 g/kg, 1.5 g/kg, 3.0 g/kg, and 4.5 g/kg). In the above four groups, a total of 1115 metabolites were identified, of which 348 (31.21%) were amino acids and their metabolites; 155 (13.9%) were organic acids and their derivatives; 133 (11.93%) were fatty acyls; 81 (7.26%) were glycerophospholipids (GPs); 80 (7.17%) were nucleotides and their metabolites; 74 (6.64%) were benzene and its substituents; 74 (6.64%) were carbohydrates and their metabolites; 59 (5.29%) were heterocyclic compounds; 52 (4.66%) were alcohols; 41 (3.68%) were amines; 21 were aldehydes, ketones, and esters (1.88%); 20 were bile acids (1.79%); 18 were coenzymes and vitamins (1.61%); 13 were hormones and hormone-related compounds (1.17%); 5 were glycerol lipids (GL) (0.45%); 4 were glycolipids (SL) (0.36%); 9 were other types of metabolites (including betaine, hydroxyurea, Kojibiose, α-tropine, etc.) (0.81%); and 2 were tryptamines, cholines, and pigments (0.18%) (Figure 3A). The total screened metabolites were clustered and visualized in a heat map (Figure 3B). The analyses of muscle metabolites from the experimental and control groups identified both common and distinct metabolic products with notable variations in the metabolite content across different groups.

#### 3.3.2. Multivariate Data Analysis and Identification of Characteristic Metabolites Between Groups

**Figure 4:**
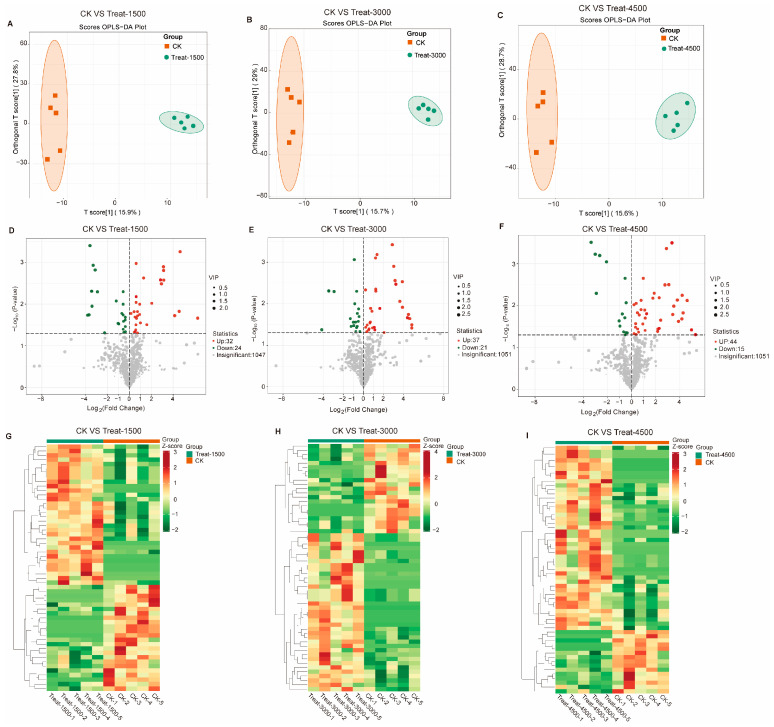
Effect of different levels of MBEs added to diet on metabolites of broiler breast muscle compared to control group. OPLS-DA analysis between CK and Treat-1500 (**A**), CK and Treat-3000 (**B**), and CK and Treat-4500 groups (**C**). Volcano plot analysis between CK and Treat-1500 (**D**), CK and Treat-3000 (**E**), and CK and Treat-4500 groups (**F**). Heat map analysis between CK and Treat-1500 (**G**), CK and Treat-3000 (**H**), and CK and Treat-4500 groups (**I**).

**Figure 5:**
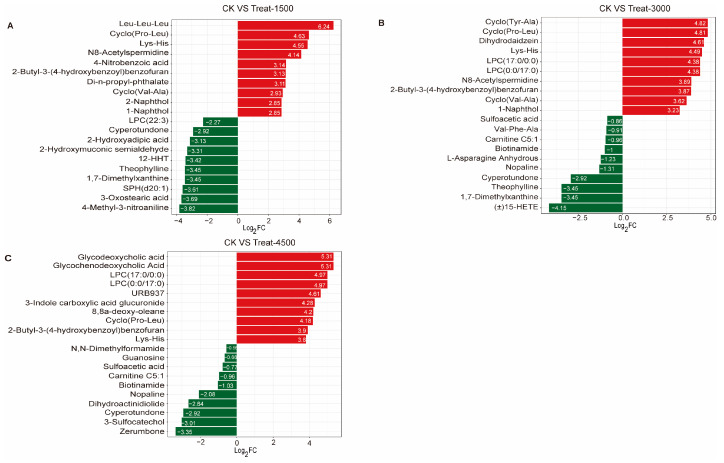
Top 10 metabolites with largest multiplicative upregulated and down-regulated adjustments. (**A**) Control group vs. Treat-1500 group. (**B**) Control group vs. Treat-3000 group. (**C**) Control group vs. Treat-4500 group. Red bars: up-regulation; Green bars: down-regulation.

**Figure 6:**
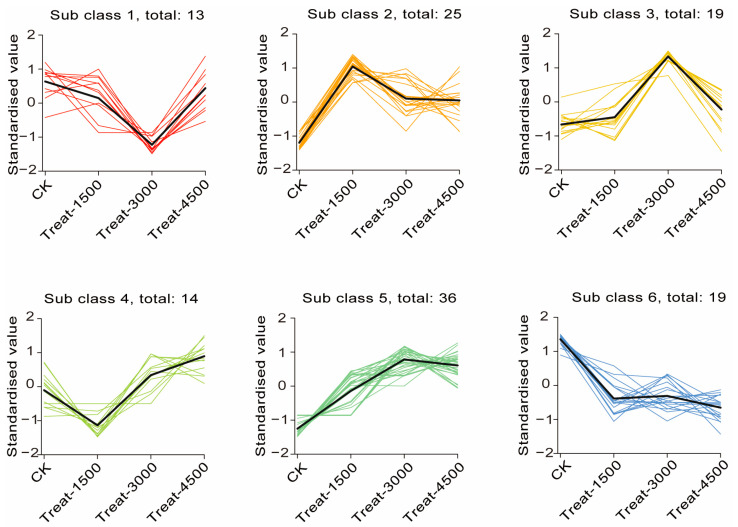
K-means clustering of differential metabolite profiles.

An Orthogonal Partial Least Squares Discriminant Analysis (OPLS-DA) was performed to validate the clustering and further explore potential biomarkers following intervention with MBEs (Figure 4A–C). The analysis revealed a significant separation between the MBE-treated group and the control group, indicating that the addition of MBEs influenced muscle metabolism. Based on the OPLS-DA results, metabolites with VIP > 1 or *p* < 0.05 were identified as differentially significant metabolites (DEMs), which were illustrated in volcano plots (Figure 4D–F). In the comparison between the control group (CK, 0 g/kg) and Treat-1500 (1.5 g/kg) group, a total of 56 DEMs were detected, comprising 32 upregulated and 24 down-regulated metabolites. In the comparison of the CK and Treat-3000 (3.0 g/kg) groups, 58 DEMs were identified, with 37 upregulated and 21 down-regulated metabolites. Additionally, in the comparison between the CK and Treat-4500 (4.5 g/kg) groups, a total of 59 DEMs were identified, consisting of 44 upregulated and 15 down-regulated metabolites. Subsequently, the relative quantitative values of all DEMs obtained from the comparison of each treatment group with the CK group were normalized and subjected to a hierarchical clustering analysis. The results indicate that the metabolite content clusters varied greatly among different groups (Figure 4G–I). Following qualitative and quantitative analyses of the detected metabolites, the top 10 metabolites were identified in descending order based on absolute fold change (FC) values, highlighting significant differences in upregulation versus down-regulation between the two groups (Figure 5). The addition of MBEs at a low dose of 1.5 g/kg significantly increased the content of several amino acids and their derivatives while simultaneously reducing the abundance of the oxidized lipid 12-HHT and oxidatively damaged low-density lipoproteins (LPCs) compared to control group. When the dosage of MBEs increased to 3.0 g/kg, a further decrease was observed in the oxidized lipid (±)15-HETE and carnitine (C5:1) alongside an increase in the concentration of small peptides derived from related amino acids. When the dosage of MBEs increased to 4.5 g/kg, there was a significant increase in the concentrations of amino acids and their derivatives, as well as glycine goose deoxycholic acid and glycine deoxycholic acid. Conversely, this increase in dosage led to significant decreases in carnitine (C5:1) and biotinamide. To investigate the trends in the relative content of metabolites across different groups, the relative content of all DEMs identified based on the established screening criteria was subjected to unit variance scaling (UV) standardization. Subsequently, a K-means clustering algorithm was applied to categorize the DEMs into six groups based on their accumulation patterns (Figure 6), with clusters 5 and 6 exhibiting significant trends. An analysis of these two clusters revealed that the metabolites in cluster 5 (Table S2) increased with the amount of addition but decreased when the dosage increased to 4.5 g/kg, although their levels were still higher than those in the control group. In cluster 6 (Table S3), the carnitine C5:1 level decreased with increasing dosages of MBEs. Additionally, the signature oxidized lipid product (±)15-HETE displayed a declining trend as the addition level was below 3.0 g/kg.

#### 3.3.3. Effects of MBEs on Metabolic Pathways in Broiler Breast Muscle

**Figure 7:**
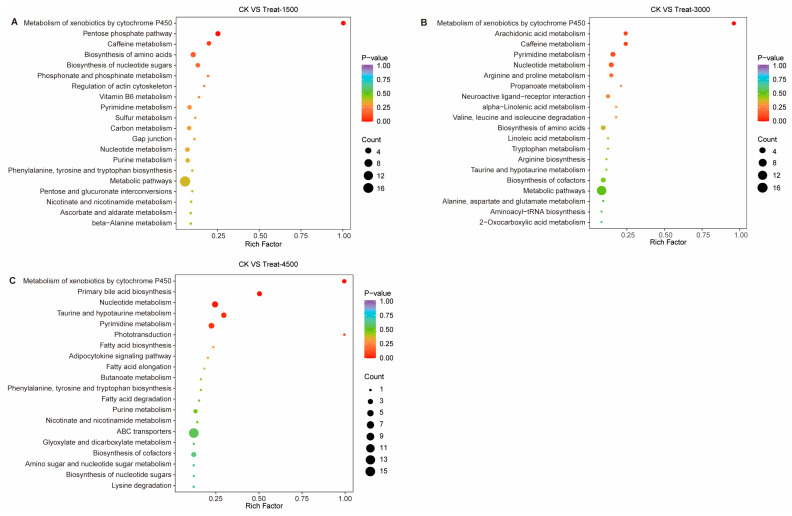
KEGG metabolic pathway enrichment analysis based on significant differential metabolites. (**A**) Control group vs. Treat-1500 group. (**B**) Control group vs. Treat-3000 group. (**C**) Control group vs. Treat-4500 group.

To elucidate the metabolic pathway of MBEs in broiler breast muscle, a KEGG enrichment analysis was conducted on the DEMs (Figure 7). All DEMs from the various comparison groups were aligned with the KEGG database to identify the pathways enriched with these metabolites. The DEMs in the CK vs. Treat-1500 group were significantly enriched in the metabolism of xenobiotics by the cytochrome P450 pathway and the pentose phosphate pathway. In the CK vs. Treat-3000 group, the DEMs were significantly enriched in the metabolism of xenobiotics by the cytochrome P450 pathway and the arachidonic acid metabolic pathway. Additionally, the CK vs. Treat-4500 group exhibited significant enrichment in the metabolism of xenobiotics by the cytochrome P450 pathway, primary bile acid metabolism pathway, and taurine and hypotaurine metabolism pathway. In all comparative groups, significant enrichment was observed in the metabolism of xenobiotics by the cytochrome P450 pathway, suggesting that MBEs may influence the expression of CYP450 family enzymes during their functional processes. Furthermore, the enrichment of the pentose phosphate pathway, arachidonic acid metabolism pathway, and bile acid metabolism pathway is associated with carbohydrate and lipid metabolism.

## 4. Discussion

Oxidative stress disrupts cell and mitochondrial membranes, resulting in an imbalance in the regulation of pro-oxidant and antioxidant systems. This imbalance alters the normal redox state of cells, generating peroxides and free radicals that can lead to cytotoxicity. Consequently, these processes damage muscle proteins, lipids, and nucleic acids, which, in turn, trigger inflammation [15,16]. Given that muscle tissue exerts immune function by insulating systemic inflammation and protecting the proliferation of CD8^+^ T Cells [17], the dysregulation of redox balance in muscle may contribute to the development of various diseases. Furthermore, fat oxidation and protein oxidation are the primary causes of meat quality deterioration during post-slaughter storage [18]. Therefore, enhancing the antioxidant capacity of broiler muscle is essential for improving muscle nutrition and flavor, as well as for increasing economic efficiency and promoting animal welfare in livestock farming. One of the effective strategies to achieve this is through the incorporation of natural antioxidants into animal diets.

Flavonoids, a subset of the polyphenol group, are primarily utilized to promote health in both animals and humans [19]. Various plant-derived flavonoids and phenolic compounds, including resveratrol, chlorogenic acid, and pterostilbene, have been reported to be effective natural antioxidants when added to animal diets. Moreover, these compounds also have the potential to improve meat quality and enhance the antioxidant capacity of meat [20,21,22]. Among these, mulberry branch flavonoids stand out due to their rich pharmacological effects. Rutin, quercetin, resveratrol, oxyresveratrol, and mulberroside A, which are contained in mulberry branch flavonoids, have good antioxidant activity [23,24]. Studies have demonstrated that mulberry branch flavonoids possess both direct (reactive oxygen species scavenging) and indirect (iron chelation) antioxidant capacity [25,26,27]. In our previous study, MBEs showed positive effects on pre-slaughter growth performance as well as post-slaughter serum biochemistry and the meat quality of broilers. In particular, the addition of MBEs at the level of 3.0 g/kg was found to improve meat quality by reducing cooking and drip losses, as well as by enhancing meat color [28]. It has been reported that the antioxidant capacity of meat is negatively correlated with drip loss and that lipid peroxidation can compromise cell membrane integrity, which, in turn, increases drip loss and affects water migration and distribution [29]. Muscle color, a key indicator of meat freshness, transitions from bright red to reddish-brown as oxygenated myoglobin oxidizes to metmyoglobin [30]. The addition of exogenous antioxidants helps protect lipids from oxidative stress, thereby stabilizing the content of oxygenated myoglobin [8]. Based on these observations, we propose that the flavonoids and polyphenols present in MBEs may be responsible for their antioxidant effects. On this basis, we further investigated the impact of MBEs on muscle antioxidant capacity. Studies have indicated that incorporating bamboo leaf flavonoids into the diets of broiler chickens reduces serum MDA levels and enhances antioxidant enzyme activities in breast muscle [31]. When flavonoid-rich mulberry leaf extracts were incorporated into the diet, serum antioxidant enzyme activities exhibited an increasing trend [32]. Furthermore, previous studies showed that aqueous extracts of Mori Ramulus significantly alleviated H_2_O_2_-induced oxidative damage in C2C12 mouse myoblasts, demonstrating that MBEs can maintain mitochondrial function and effectively scavenge reactive oxygen species [33].

Mulberry leaf extracts effectively protected mouse hepatocytes from oxidative stress in a dose-dependent manner. This protective effect was associated with the upregulation of total superoxide dismutase (T-SOD) and total antioxidant capacity (T-AOC) as well as a reduction in reactive oxygen species (ROS) levels. Furthermore, the amelioration of oxidative stress induced by mulberry leaf extracts was found to be mediated through the Nrf2 signaling pathway [34]. In addition, curcumin’s antioxidant capacity was shown to activate the Nrf2-antioxidant response element to protect the ileum, as demonstrated in another study investigating the effects of curcumin on aflatoxin B1-induced ileal damage in ducks [27]. In our study, the addition of MBEs significantly enhanced the expression of *Nrf2* and its downstream antioxidant genes, including *CAT*, *GSH-Px*, *SOD1*, *GCLC*, *GCLM*, *HO-1*, and *NQO-1*, in breast muscle. A related study identified a positive correlation between antioxidant enzyme activity, the expression of *Nrf2* and its downstream genes, and various meat quality parameters, including color, tenderness, water retention, and nutrient type and content [21]. Therefore, it is hypothesized that MBEs may enhance the antioxidant capacity of muscle by activating the Nrf2 signaling pathway, ultimately contributing to improving meat quality.

Metabolomics, an emerging field in molecular biology, is increasingly utilized for the thorough analysis of metabolites in meat, exploring the effects of feed additives on meat, and investigating flavor changes in meat during storage and processing [35,36,37]. Changes in metabolic profiles provide insights into the mechanisms underlying meat quality responses to stress or nutritional factors. The widely targeted metabolomics combines the advantages of both untargeted and targeted metabolomics, achieving precise quantification while also offering broad coverage [38]. Therefore, we chose widely targeted metabolomics to investigate the effects of MBEs on muscle metabolites.

In this study, the most abundant and diverse metabolites detected were amino acids and their derivatives, aligning with the prior study that also recognized them in lamb meat quality assessments [39]. After the addition of MBEs, the most significantly upregulated metabolites among the top 20 with the highest fold changes compared to the control group were small peptides (dipeptides and tripeptides). This suggested that, after slaughter, intramyocardial peptidases and exopeptidases degraded muscle proteins, resulting in a complex mixture of small peptides during the degradation process [40]. Small bioactive peptides with specific sequences, such as those containing proline, tyrosine, and methionine, exhibited anti-hypertensive activity. Additionally, small peptides containing histidine, proline, and leucine demonstrated antioxidant capacity [41]. Notably, small peptides of less than 5 kDa were more effective at scavenging 2,2-diphenyl-1-picrylhydrazyl (DPPH) compared to larger peptides [42]. An analysis of our results revealed a significant increase in the contents of various small peptides containing histidine, lysine, and leucine compared to the control group. The increases in these small peptides suggested their potential role in antioxidant capacity. A previous study demonstrated that dietary leucine supplementation enhances antioxidant enzyme activities and promotes antioxidant gene expression in piglets [43]. Furthermore, leucine has been shown to positively modulate antioxidant capacity and immune competence in early weaned adult rats [44]. Proline serves as a regulator of stress and cellular bioenergetics, with supplementation shown to enhance redox status [45]. Histidine not only decreases the levels of H_2_O_2_ and MDA but also boosts the activity of antioxidant enzymes. Additionally, it enhances the antioxidant capacity of the glutathione system by increasing the levels of glutamate and cysteine [46].

**Figure 8:**
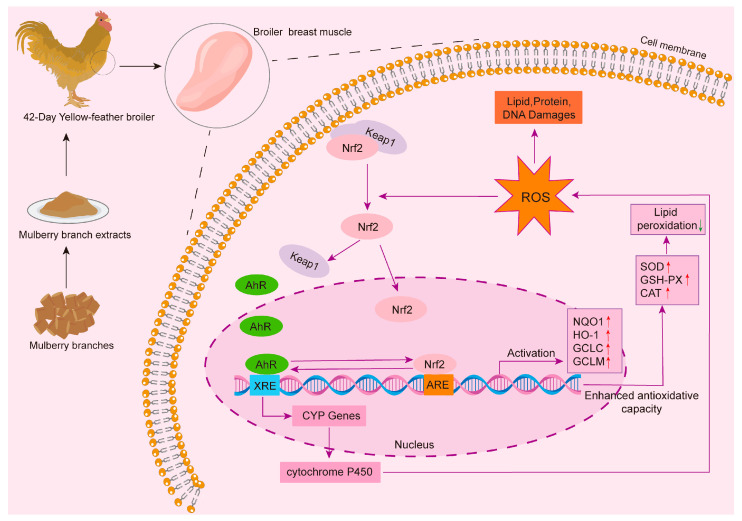
Schematic plot of MBEs ameliorating antioxidant capacity of broiler breast muscle. XRE: xenobiotic response element; AhR: aryl hydrocarbon receptor; ARE: antioxidant response element. Red arrows: up-regulation; Green arrows: down- regulation.

This study demonstrated that the addition of MBEs may enhance antioxidant capacity by modifying the amino acid metabolic profile of broiler muscle. This process could lead to an increase in various amino acids with antioxidant capacity and their corresponding small peptides, thereby potentially improving the nutritional quality and tenderness of broiler breast muscle. A KEGG enrichment analysis of the DEMs revealed that the significantly enriched metabolic pathways included the metabolism of xenobiotics by the cytochrome P450 pathway, the pentose phosphate pathway, the arachidonic acid metabolic pathway, and primary bile acid biosynthesis pathway. Among these pathways, it is important to highlight that the metabolism of xenobiotics by cytochrome P450 (CYP450) was the common pathway enriched across all three treatment groups (Figure 8). The CYP450 metabolic pathway primarily operates through cytochrome P450 enzymes. CYP450 enzymes play a prominent role in the phase I metabolism of approximately 75% of drug metabolism reactions, and uncoupling occurs when CYP450 enzymes catalyze the drug, resulting in the production of ROS [47,48,49]. The binding of hazardous substances to the aryl hydrocarbon receptor (AhR) activates the AhR signaling pathway, which subsequently activates the expression of CYP450 enzymes and then generates ROS that indirectly activate the expression of Nrf2, resulting in the increased expression of Nrf2 pathway genes and antioxidant enzymes [50,51]. Overall, MBEs can enhance antioxidant capacity through the direct activation of Nrf2 as well as the indirect activation of the AhR signaling pathway, which triggers the activation of CYP450 enzymes. Additionally, Nrf2 plays a crucial role in the negative regulation of adipocyte differentiation and inhibits fat formation by modulating the expression of AhR and downstream target genes, including CYP metabolic enzymes. This mechanism may also represent a significant aspect of how MBEs exert antioxidant effects.

## 5. Conclusions

In conclusion, the present study demonstrated that the inclusion of MBEs in the diet enhanced muscle antioxidant capacity by increasing the expression of genes on the Nrf2 signaling pathway and the activities of antioxidant enzymes. Metabolic pathway analyses indicated that the DEMs were primarily enriched in the metabolism of xenobiotics by the cytochrome P450 pathway, the pentose phosphate pathway, the arachidonic acid metabolic pathway, and the primary bile acid biosynthesis pathway. 12-HTT, (±)15-HETE, and various small peptides were identified as potential biomarkers of MBEs, which serve as antioxidants for muscle protection. These findings enhance our understanding of the antioxidant defense of MBEs in yellow-feather broilers, facilitating the development of natural, effective, and safe antioxidants to improve muscle antioxidant capacity and even promote animal welfare in intensive poultry farming. However, future studies should focus on isolating the individual components of MBEs and assessing their effects on livestock farming or other industries.

## Figures and Tables

**Table 1:** Compositions and nutritional levels of basic diets.

Items	42–65 Days of Age	66–90 Days of Age
Ingredients		
Corn	58.06	62
Soybean meal (crude protein 43%)	26	19.9
Fish meal	2	2
DDGS (distilled dried grains with solubles)	3	5
Wheat bran	4	-
WMAR (Wheat Middling and Red Dog)	-	4.16
Soybean oil	2	2.5
Limestone	1.2	1
CaHPO_4_	1.5	1.2
NaCl	0.24	0.24
Premix ^1^	2	2
Total	100	100
Nutrient levels ^2^		
Metabolic energy, MJ/kg	12.1	12.66
Crude protein	18.51	16.77
Calcium	0.98	0.82
Total phosphorus	0.67	0.58
Methionine + cysteine	0.7	0.64
Lysine	0.96	0.82
Methionine	0.32	0.30

^1^ The premix provided the following (per kilogram of diet): VA, 9000 IU; VD3, 500 IU; VE, 35 IU; VK, 32.2 mg; VB12, 15 mg; VB6, 50 mg; niacin, 35 mg; pantothenic acid, 10 mg; folic acid, 1 mg; thiamine, 3 mg; riboflavin, 5 mg; biotin, 0.5 mg; choline, 1000 mg; copper, 7 mg; Iron, 80 mg; zinc, 60 mg; manganese, 60 mg; iodine, 0.7 mg; Se, 0.15 mg. ^2^ Crude protein, calcium, and total phosphorus are measured values, while the others are calculated values.

**Table 2:** The primers used for quantitative PCR.

GeneBank ID	Name	Primer Sequence (5′-3′)
NM_205518	*β-actin*	F: GCCAACAGAGAGAAGATGACAC R: GTAACACCATCACCAGAGTCCA
NM_205117.1	*Nrf2*	F: AGGAAGAAGGTGCTTTTCGCA R: TCTGTTCCTCTTCACTGCCAC
NM_001163245.1	*GSH-Px*	F: CAAAGTTGCGGTCAGTGGA R: AGAGTCCCAGGCCTTTACTACTTTC
NM_205064.1	*SOD1*	F: TTGTCTGATGGAGATCATGGCTTC R: TGCTTGCCTTCAGGATTAAAGTGA
NM_001031215.2	*CAT*	F: GGTTCGGTGGGGTTGTCTTT R: CACCAGTGGTCAAGGCATCT
NM_001277620.2	*NQO1*	F: TCGCCGAGCAGAAGAAGATTGAA R: CGGTGGTGAGTGACAGCATGG
NM_205344.2	*HO-1*	F: ACGAGTTCAAGCTGGTCACG R: GGATGCTTCTTGCCAACGAC
XM_046915268.1	*GCLC*	F: TCTGTAGATGATCGAACGC R: TCCTTTATTAGGTGCTCGTAG
NM_001007953.2	*GCLM*	F: GCTGCTAACTCACAATGACC R: TGCATGATATAGCCTTTGGAC

*Nrf2*: nuclear factor erythroid 2-related factor 2; *GSH-Px*: glutathione peroxidase; *SOD1*: superoxide dismutase 1; *CAT*: catalase; *HO-1*: heme oxygenase-1; *NQO-1*: NAD(P)H quinone oxidoreductase-1; *GCLC*: glutamyl–cysteine ligase; *GCLM*: glutamate–cysteine ligase modifier subunit.

## Data Availability

The datasets supporting the conclusions of this article are included within the article.

## Supplementary Materials

The following supporting information can be downloaded at https://www.mdpi.com/article/10.3390/ani14243702/s1, Table S1: Quantitative data on the relative expression of antioxidant enzymes and antioxidant genes; Table S2: Metabolite information clustered into Cluster 5; Table S3: Metabolite information clustered into Cluster 6.
