# Supplementary material for: Mulberry Branch Extracts Enhance the Antioxidant Capacity of Broiler Breast Muscle by Activating the Nrf2 and Cytochrome P450 Signaling Pathway
Source: Animals (Basel). 2024 Dec 22;14(24):3702. doi: 10.3390/ani14243702 (PMC11672785; doi:10.3390/ani14243702)
Supplement: Supplementary file 1 [file animals-14-03702-s001.zip › Table S1.pdf]

Table S1. Quantitative data on the relative expression of antioxidant enzymes and antioxidant genes

Relative mRNA expression level

Parameters	CK	Treat-1500	Treat-3000	Treat-4500
**GSH-Px**	0.89±0.13[c]	1.34±0.28[bc]	2.38±0.4[a]	1.73±0.27[b]
**SOD**	1.28±0.25[b]	1.55±0.37[b]	2.55±0.50[a]	1.70±0.57[b]
**CAT**	0.56±0.11[c]	0.87±0.40[bc]	1.44±0.28[a]	1.32±0.28[ab]
**Nrf2**	0.83±0.06[c]	0.90±0.08[c]	1.73±0.18[a]	1.45±0.08[b]
**HO-1**	1.09±0.13[c]	1.35±0.16[bc]	1.60±0.18[b]	2.00±0.37[a]
**NQO-1**	0.94±0.12[b]	1.00±0.24[b]	1.73±0.16[a]	1.88±0.31[a]
**GCLC**	1.28±0.26[b]	1.55±0.37[b]	2.21±0.32[a]	1.42±0.08[b]
**GCLM**	0.56±0.11[b]	0.87±0.40[b]	1.44±0.17[a]	1.09±0.45[a]

Muscle antioxidative status measurements

Parameters	CK	Treat-1500	Treat-3000	Treat-4500
**T-SOD(U/mL)**	143.33±29.592[d]	250.55±6.786[b]	303.01±20.464[a]	208.05±35.340[c]
**MDA(nmol/mL)**	4.94±0.41[a]	3.11±0.45[c]	2.94±0.44[c]	4.16±0.4[b]
**T-AOC (U/mL)**	13.18±2.185[c]	21.18±3.235[b]	26.16±2.485[a]	18.47±1.911[b]
**GSH-Px (U/mL)**	260.79±34.548[c]	380.56±58.186[b]	459.26±21.097[a]	333.04±41.623[b]
**CAT(U/mL)**	48.43±7.212[c]	75.73±9.136[a]	81.78±9.421[a]	61.40±8.757[b]
