# Supplementary material for: Mulberry Branch Extracts Enhance the Antioxidant Capacity of Broiler Breast Muscle by Activating the Nrf2 and Cytochrome P450 Signaling Pathway
Source: Animals (Basel). 2024 Dec 22;14(24):3702. doi: 10.3390/ani14243702 (PMC11672785; doi:10.3390/ani14243702)
Supplement: Supplementary file 1 [file animals-14-03702-s001.zip › Table S2.pdf]

Table S2. Metabolite information clustered into Cluster 5

Formula	Compounds	Class I	Ionization model
C4H6N2O2	1-Methyl-hydantoin	Heterocyclic compounds	[M+H]+
C10H8O	1-Naphthol	Benzene and substituted derivatives	[M-H]-
C4H11NO2	2-Amino-2-methyl-1,3-propanediol	Alcohol and amines	[M+H]+
C19H18O3	2-Butyl-3-(4-hydroxybenzoyl)benzofuran	Benzene and substituted derivatives	[M+H]+
C3H6O3	2-Methoxyacetic acid	Organic acid and Its derivatives	[M-H]-
C10H8O	2-Naphthol	Benzene and substituted derivatives	[M-H]-
C10H13O10P	5-O-(1-carboxyvinyl)-3-phosphate	Organic acid and Its derivatives	[M-H]-
C9H19N5O3	Ala-Arg	Amino acid and Its metabolites	[M+H]+
C12H16N2O3	Ala-Phe	Amino acid and Its metabolites	[M+H]+
C8H15N3O4	Ala-gln	Amino acid and Its metabolites	[M+H]+
C9H19N5O3	Arg-Ala	Amino acid and Its metabolites	[M+H]+
C12H25N5O3	Arg-Leu	Amino acid and Its metabolites	[M+H]+
C9H15N3O5	Asn-Hyp	Amino acid and Its metabolites	[M+H]+
C4H4N2O3	Barbituric acid	Heterocyclic compounds	[M+H]+
C9H15N3O11P2	Cytidine 5'-diphosphate	Nucleotide and Its metabolites	[M+Na]+
C15H12O4	Dihydrodaidzein	Benzene and substituted derivatives	[M-H]-
C9H18O2	FFA(9:0)	FA	[M-H]-
C26H43NO5	Glycochenodeoxycholic Acid	Bile acids	[M-H]-
C26H43NO5	Glycodeoxycholic acid	Bile acids	[M-H]-
C10H14N5O8P	Guanosine-5'-monophosphate	Nucleotide and Its metabolites	[M-H]-
C11H21N3O4	Hyp-Lys	Amino acid and Its metabolites	[M-H]-
C7H11NO5	L-2-amino-6-oximelic acid	Organic acid and Its derivatives	[M-H]-
C8H15NO6	N-Acetyl-D-Glucosamine	Carbohydrates and Its metabolites	[M-H]-
C7H11NO5	N-Acetyl-L-Glutamic Acid	Amino acid and Its metabolites	[M-H]-
C11H13NO3	N-acetyl-D-phenylalanine	Amino acid and Its metabolites	[M+H]+
C8H16N4O3	Nα-Acetyl-L-Arginine	Amino acid and Its metabolites	[M-H]-
C12H16N2O3	Phe-Ala	Amino acid and Its metabolites	[M+H]+
C7H13NO2	Proline betaine	Amino acid and Its metabolites	[M+H]+
C26H45NO7S	Tauro-beta-muricholic acid	Bile acids	[M-H]-
C26H45NO7S	Taurocholic acid	Bile acids	[M-H]-
C26H45NO7S	Taurohyocholic acid	Bile acids	[M-H]-
C12H16N2O4	Tyr-Ala	Amino acid and Its metabolites	[M+H]+
C14H22N2O16P2	UDP-xylose	Carbohydrates and Its metabolites	[M-H]-
C20H22N2O4	URB937	Benzene and substituted derivatives	[M+H]+

C9H13N2O9P	Uridine 5-Monophosphate	Nucleotide and Its metabolites	[M-H]-
C9H14N2O12P2	Uridine 5′-Diphosphate	Nucleotide and Its metabolites	[M-H]-
