# Supplementary material for: Mulberry Branch Extracts Enhance the Antioxidant Capacity of Broiler Breast Muscle by Activating the Nrf2 and Cytochrome P450 Signaling Pathway
Source: Animals (Basel). 2024 Dec 22;14(24):3702. doi: 10.3390/ani14243702 (PMC11672785; doi:10.3390/ani14243702)
Supplement: Supplementary file 1 [file animals-14-03702-s001.zip › Table S3.pdf]

Table S3. Metabolite information clustered into Cluster 6

Formula	Compounds	Class I	Ionization model
C6H10O5	1,6-anhydro-β-D-glucose	Carbohydrates and Its metabolites	[M-H]-
C9H12N2O5	4-Deoxyuridine	Nucleotide and Its metabolites	[M+H]+
C5H6O6	4-Hydroxy-2-Oxoglutaric Acid	Organic acid and Its derivatives	[M-H]-
C10H17N3O2S	Biotinamide	Alcohol and amines	[M+H]+
C12H21NO4	Carnitine C5:1	FA	[M+H]+
C15H22O	Cyperotundone	Aldehyde,Ketones,Esters	[M+H]+
C10H13N5O5	Guanosine	Nucleotide and Its metabolites	[M-H]-
C14H30O8	Heptethylene-glycol	Alcohol and amines	[M+H]+
C4H8N2O3	L-Asparagine Anhydrous	Amino acid and Its metabolites	[M-H]-
C30H56NO7P	LPC(22:3)	GP	[M+H]+
C5H6O4	Monomethyl Fumarate	Aldehyde,Ketones,Esters	[M-H]-
C3H7NO	N,N-Dimethylformamide	Alcohol and amines	[M+H]+
C16H31NO3	N-Myristoylglycine	Amino acid and Its metabolites	[M-H]-
C11H20N4O6	Nopaline	Amino acid and Its metabolites	[M+H]+
C9H15N3O4	Pro-Asn	Amino acid and Its metabolites	[M+H]+
C9H14N2O5	Pro-Asp	Amino acid and Its metabolites	[M+H]+
C10H16N2O4	Proline-Hydroxyproline	Amino acid and Its metabolites	[M+H]+
C2H4O5S	Sulfoacetic acid	Organic acid and Its derivatives	[M-H]-
C15H22O	Zerumbone	Aldehyde,Ketones,Esters	[M+H]+
